# Genetics and Functional Mechanisms of STAT3 Polymorphisms in Human Tuberculosis

**DOI:** 10.3389/fcimb.2021.669394

**Published:** 2021-07-07

**Authors:** Feifei Wang, Guixian Huang, Ling Shen, Ying Peng, Wei Sha, Zheng W. Chen, Hongbo Shen

**Affiliations:** ^1^ Clinic and Research Center of Tuberculosis, Shanghai Key Laboratory of Tuberculosis, Shanghai Pulmonary Hospital, Institute for Advanced Study, Tongji University School of Medicine, Shanghai, China; ^2^ Key Laboratory of Medical Molecular Virology (MOE/NHC/CAMS), Department of Medical Microbiology and Parasitology, School of Basic Medical Sciences, Shanghai Medical College, Fudan University, Shanghai, China; ^3^ Department of Microbiology & Immunology and Center for Primate Biomedical Research, University of Illinois College of Medicine, Chicago, IL, United States

**Keywords:** tuberculosis severity, STAT3, polymorphisms, anti-mycobacterial pathways, VDR-related pathway

## Abstract

Signal transducer and activator of transcription-3 (STAT3) plays an important role in biological balance. Our and others previous studies implied that STAT3 had a great effect on fast-acting innate immunity against tuberculosis (TB). We hypothesized that *stat3* SNP down-regulation of STAT3 leads to a change in susceptibility to TB in humans. To test this hypothesis, we investigated STAT3 SNPs using SNP scan™ technique in a case-control study of TB patients (n = 470) and HC subjects (n = 356), and then conducted functional studies of them using cellular models. We found that SNPs in STAT3 3`-UTR of rs1053004 TT and rs1053005 AA genotypes or T-A haplotype were associated with susceptibility to TB or TB severity. While the TT/AA genotype correlated with the low constitutive expression of *stat3* and *IL-17A* in PBMC, the variant *stat3* of rs1053004-rs1053005 T-A haplotype indeed reduced *stat3* expression in reporter assays. Interestingly, host PBMC expressing the rs1053005 AA genotype and low constitutive *stat3* exhibited the reduced ability to mount fast-acting innate immunity against mycobacterial infection in cellular models. Finally, mechanistic experiments showed that the STAT3 down-regulation broadly depressed STAT3 downstream anti-mycobacterial activities involving VDR-related CAMP pathway as well as IL-32, iNOS and autophagy mechanisms, leading to an enhanced mycobacterial infection. The findings of this study suggest that low constitutive stat3 derived from the TT/AA genotype/T-A haplotype acts to down-regulate STAT3, depressing multiple anti-mycobacterial pathways/mechanisms downstream, which leads to an enhanced mycobacterial infection or TB in high-risk individuals.

## Introduction

Tuberculosis (TB), caused by *Mycobacterium tuberculosis* (Mtb), is one of the top 10 causes of death worldwide and a leading killer among infectious diseases. Around 10 million people fell ill with TB, and 1.2 million died from the disease in 2018. After exposure to TB pathogen aerosol, host immune factors are decisive for a potential clinical outcome of resulting active TB, latent TB infection (LTBI) or resisters with early clearance of TB bacillus without immune signature of infection (Simmons et al., 2018).

Signal transducers and activators of transcription-3 (STAT3) are widely expressed in host cells and have been shown to play multiple and distinct biological roles in regulating immune balance (Hillmer et al., 2016). STAT3, as acute-phase response factor, displays single nucleotide polymorphisms (SNP) or aberrant expression in some selected human populations. It has been reported that STAT3 SNP were significantly associated with cancers, immunodeficiency, autoimmunity and viral hepatitis (Hong et al., 2016). SNP rs1053004 in 3’-UTR of STAT3 was associated with a reduced risk of pancreatic cancer (Zhu et al., 2016). STAT3 rs1905341 was associated with better response to IFN-α in patients with metastatic renal cell carcinoma, serving as a potential predictive marker for treatment with IFN-α (Eto et al., 2013). STAT3 SNP (rs744166) was associated with multiple sclerosis (MS), whereas the protective haplotype for MS in STAT3 is a risk allele for Crohn’s disease, implying that STAT3 represents a shared risk locus for at least two autoimmune diseases (Jakkula et al., 2010). Furthermore, aberrantly expressed STAT3 was also associated with viral hepatitis. SNP rs1053004 genotype CC and the rs1053005 genotype GG were more frequent in patients with chronic hepatitis B virus (HBV) infection than in healthy controls. The rs1053004-rs1053005 haplotype T-G was less frequent in patients with chronic HBV infection than in healthy controls (Li et al., 2018). Nevertheless, the reported STAT3 SNPs have not been characterized for an altered susceptibility or immunity *via* in-depth mechanistic studies of human diseases.

Correlation between STAT3 SNP and susceptibility to TB has not been demonstrated, although STAT3 has been reported to regulate the development and function of T helper 17 (Th17) cells for anti-TB cellular immune responses (Ernst, 2018). In fact, STAT3 plays essential roles in differentiating both the adaptive CD4+ Th17 subset and the unconventional T subset such as γδ T cells (Wilson et al., 2015; Shen et al., 2017). Th17 and microbe-specific γδ T subsets appear to be required for host defense against Mtb infection (Khader et al., 2007; Shen et al., 2019). Despite the role of STAT3 in T-cell functions, STAT3 and its downstream innate pathways have not been well-defined in fast immunity and protective mechanisms in human TB and other infections. Given the broad biological roles of STAT3 (Hillmer et al., 2016), we hypothesized that STAT3 and relevant downstream pathways play an important role in fast-acting innate immunity against TB, and that *stat3* SNP down-regulation of STAT3 and downstream pathways leads to a change in susceptibility to TB in humans.

To test our hypothesis, we performed the STAT3 SNP analysis in humans and then conducted in-depth mechanistic experiments using cellular models. Our experimental studies in humans and cellular models support the hypothesis and provides previously unreported findings and potential mechanisms regarding molecular genetics and functions of STAT3 SNP in human TB.

## Materials and Methods

### Human Subjects and SNP Genotypes Analysis

The study was approved by the institutional review boards for human subjects’ research and institutional biosafety committees at the Shanghai Pulmonary Hospital (SPH) of Tongji University. All subjects are adults, and signed written informed consent.

TB patients were recruited at the Shanghai Pulmonary Hospital (Shanghai, China). The severe TB and mild TB patients were classified according to our previous study (Fan et al., 2017). Briefly, all active TB patients were confirmed by bacteriology or pathology. According to the chest computed tomography (CT) scan results, severe TB patients were classified with at least one large cavity of ≥3 cm in diameter or at least three cavities regardless of the diameter of cavities, mild TB patients had mild lesions in ≤2 lung fields or non-cavitary lesions in lungs. Age- and sex-matched uninfected volunteers without clinical and immunological evidence of TB or latent TB were recruited as healthy control (HC). All participants were tested for human immunodeficiency virus (HIV), hepatitis C virus (HCV) and HBV. Individuals with HIV, HCV, HBV infection and other infectious diseases or cancers were excluded.

Whole blood samples were collected from enrolled subjects and used to isolate genomic DNA using the QIAamp DNA Blood Mini kit (Qiagen, Hilden, Germany). SNP sites were selected from previous reports, by functional relevance and haplotype-tagging capacity as indicated on the hapmap website (www.hapmap.org). SNP genotypes were determined by SNP scan TM kits (Genesky Biotechnologies Inc., Shanghai, China). The collaborative genetic studies of most subjects at SPH and others were completed.

### Peripheral Blood Mononuclear Cell (PBMC) Isolation and Real-Time Quantitative Polymerase Chain Reaction (q-PCR) Analysis for Gene Expression

PBMCs were isolated from ethylenediaminetetraacetic acid (EDTA)-treated blood of human subjects using Ficoll–Paque plus density gradient centrifugation and then cultured with RPMI1640 media supplemented with 2 mM glutamine, 50 U/ml of penicillin and 50 μg/ml of streptomycin, and containing 10% FBS (Invitrogen) according to our previous publications (Chen et al., 2012; Shen et al., 2017).

Total RNA was extracted from human PBMCs using RNA column enrichment procedures (Zymo Research, CA). CD14+ and Vδ2+ T cells were isolated and enriched from fresh PBMC using MACS methods (Miltenyi Biotec, German), respectively. RNA was reverse-transcribed into complementary DNA (cDNA). The cDNA was used to amplify target gene fragment in triplicate reactions for each gene. Sequences of q-PCR primers were listed in **Table 1**. The β-actin was used as internal control gene for normalization.

**Table 1:** Primers used for qPCR.

Gene	Forward primer	Reverse primer
STAT3	5′-TTTGAGACCGAGGTGTATCACC-3′	5′-GGTCAGCATGTTGTACCACAGG-3′
SOCS3	5′-TTCTGATCCGCGACAGCTC-3′	5′-TGCAGAGAGAAGCTGCCCC-3′
IL-32	5′-ATGCACCAGGCCATAGAAAG-3′	5′-CGGCACCGTAATCCATCTC-3′
VDR	5′-CTGACCCTGGAGACTTTGAC-3′	5′-TTCCTCTGCACTTCCTCATC-3′
CAMP	5′-AGGATTGTGACTTCAAGAAGGACG-3′	5′-GTTTATTTCTCAGAGCCCAGAAGC-3′
CYP27B1	5′-ACC CGA CAC GGA GAC CTT C-3′	5′-CACAGGTGCGACAACTGGTA-3′
DEFB4A	5′-GGT GTT TTT GGT GGT ATA GGC G-3′	5′-AGG GCA AAA GAC TGG ATG ACA-3′
β-actin	5’-GCCCTGAGGCACTCTTCCA-3’	5’-TGTTGGCGTACAGGTCTTTGC-3’

### Dual-Luciferase Reporter Assay

A wild-type or 3’-UTR of STAT3 fragments with haplotypes of T-A, T-G and C-G of SNP rs1053004-rs1053005 were constructed and inserted downstream of the luciferase reporter gene of the miR-RB-Report vector (Ribobio, Guangzhou, China), respectively. Lipofectamine 3000 were used to transfect the reporter plasmids into 293T cells. Dual luciferase reporter system kit (Promega, USA) was used to detect firefly and renilla luciferase activity.

### Mycobacteria Strains and Culture

The *Mycobacteria bovis* Bacillus Calmette–Guerin (BCG) Danish strain (ATCC 35733) and *M. tuberculosis* H37Rv were grown at 37°C in Difco Middlebrook 7H9 broth (Becton Dickinson) or on Middlebrook 7H10 agar supplemented with 10% oleic acid-albumin-dextrose-catalase-enriched Middlebrook (OADC, BD), 0.2% glycerol and 0.05% Tween-80 for 3–4 weeks. Mycobacteria were cultured in the ABSL-II level lab of the Shanghai Pulmonary Hospital of Tongji University.

### Mycobacteria Infection of Host Cells

The human alveolar epithelial cell line A549, human macrophage THP-1 and mice macrophage RAW264.7 were grown in RPMI 1640 supplemented with L-glutamine (2 mM), sodium pyruvate (1 mM) and 10% heat-inactivated fetal bovine serum (FBS).

THP-1 cells were treated with 50 ng/ml Phorbol 12-myristate 13-actate (PMA, Sigma-Aldrich) for 48 h to differentiate into macrophages, then washed with PBS and maintained for infection.

In brief, cells were infected with BCG at a multiplicity-of-infection (MOI) of 10 for ~4 h. Cells were infected with H37Rv at a MOI of 4 for 4 h. After infection, extracellular bacilli were removed by washing with PBS four times. Then, mycobacteria-infected cells were co-cultured with naive PBMC containing monocytes/macrophages, innate-like γδ T cells and other innate lymphocytes in media without antibiotics for 3 days. Then, co-cultured PBMC with the infected monocytes/macrophages and lung cells were lysed in sterile PBS with SDS. Serials dilutions were performed for quantitative culturing. Mycobacteria viability were quantified *via* counting CFU (Yang et al., 2018).

### Western Blotting

Cells were transferred by lentivirus vector carrying STAT3 shRNA or empty lentivirus vector, and stimulated by Vitamin D (VD), BCG and medium overnight, respectively. Then, cells were lysed by incubation in RIPA lysis buffer on ice for 5 min. Next, lysates were separated by SDS-PAGE and transferred to a polyvinylidene difluoride membrane (Merck/Millipore). After blocking with 5% BSA, the membrane was incubated with Abs against STAT3 (Sangon Biotech), LC3 (Abcam), or GAPDH (Sangon Biotech) overnight at 4°C, followed by incubation with the respective secondary Abs.

### Statistical Analysis

The allele and genotype frequencies were determined by direct counting. The demographic characteristics of the different groups were compared by chi-squared test using Statistical Package for Social Sciences (SPSS) version 21.0 software (SPSS Inc., IBM, Chicago, USA). A chi-squared test was performed to investigate the associations between allele frequencies and TB, and unconditional logistic regression analysis was used to investigate the associations between genotypes and TB assuming various genetic models (dominant, recessive and additive), respectively, in case and control groups using Plink software (http://pngu.mgh.harvard.edu/~purcell/plink/) in accordance with the Hardy–Weinberg equilibrium in the controls. False discovery rate (FDR) correction of multiple hypothesis testing was performed.

Statistical analysis was done by using GraphPad Prism software (GraphPad Software, CA). Data were analyzed by the Student *t* test (parametric method) or by the Mann–Whitney test (nonparametric method).

## Results

### STAT3 SNP rs1053004 and rs1053005 Loci Exhibited Linkage Disequilibrium in TB Patients; rs1053004 TT and rs1053005 AA Genotypes or the T-A Haplotype Were Associated With an Increased Susceptibility to TB

It remains unknown whether genetic variations of STAT3 could influence the susceptibility or resistance to TB in humans. Some publications demonstrated that STAT3 SNPs appeared to be associated with cancers, autoimmunity or selected infections. However, STAT3 SNP association with TB has not been reported in humans. Here, we analyzed genetic polymorphisms of rs1053005, rs1053004, rs2293152, rs744166 using SNP scan™ technique in a case-control study of TB patients (n = 470) and HC subjects (n = 356). The genetic frequencies of four SNPs in this study were accorded with Hardy–Weinberg Equilibrium (**Table 2**), using the method previously reported (Györffy et al., 2004). Logistic regression analysis was also performed for four SNPs (**Table 3**), using the analysis models of Codominant, Dominant, Recessive and Additive superposition, as previously reported (Eto et al., 2013).

**Table 2:** Hardy–Weinberg equilibrium analysis.

SNPs	HWpval
**rs1053005**	0.3181
**rs1053004**	0.2529
**rs2293152**	0.3979
**rs744166**	0.8234

**Table 3:** Logistic regression analysis results.

SNPs	Model	Genotype	Cases (n)	n%	Controls (n)	n%	OR (95%CI)	P-value
**s2293152**	Codominant	C/C	108	23.0%	76	21.3%	-	-
		C/G	260	55.3%	185	52.0%	0.989 (0.6978-1.402)	0.9504
		G/G	78	16.6%	84	23.6%	0.6534 (0.427-1)	0.05003
	Dominant	C/C	108	23.0%	76	21.3%	0.8842 (0.6329-1.235)	0.4706
		C/G-G/G	338	71.9%	269	75.6%		
	Recessive	C/C-C/G	368	78.3%	261	73.3%	0.6586 (0.4657-0.9314)	0.01819
		G/G	78	16.6%	84	23.6%		
	Additive	–	–		–		0.8123 (0.656-1.006)	0.05668
**rs1053005**	Codominant	A/A	231	49.1%	149	41.9%	-	-
		A/G	185	39.4%	160	44.9%	0.7458 (0.5551-1.002)	0.05156
		G/G	51	10.9%	45	12.6%	0.731 (0.4658-1.147)	0.173
	Dominant	A/A	231	49.1%	149	41.9%	0.7426 (0.5622-0.9808)	0.03603
		A/G-G/G	236	50.2%	205	57.6%		
	Recessive	A/A-A/G	416	88.5%	309	86.8%	0.8418 (0.5492-1.29)	0.4294
		G/G	51	10.9%	45	12.6%		
	Additive	–	–		–		0.8197 (0.6687-1.005)	0.05561
**rs1053004**	Codominant	T/T	194	41.3%	129	36.2%	-	-
		T/C	162	34.5%	154	43.3%	0.6995 (0.5113-0.957)	0.02542
		C/C	53	11.3%	52	14.6%	0.6777 (0.4353-1.055)	0.08499
	Dominant	T/T	194	41.3%	129	36.2%	0.694 (0.5175-0.9307)	0.01469
		T/C-C/C	215	45.7%	206	57.9%		
	Recessive	T/T-T/C	356	75.7%	283	79.5%	0.8102 (0.536-1.225)	0.3182
		C/C	53	11.3%	52	14.6%		
	Additive	–	–		–		0.7906 (0.6427-0.9725)	0.02619
**rs744166**	Codominant	T/T	182	38.7%	120	33.7%	-	-
		T/C	206	43.8%	181	50.8%	0.7504 (0.5532-1.018)	0.06496
		C/C	61	13.0%	47	13.2%	0.8557 (0.5485-1.335)	0.4924
	Dominant	T/T	182	38.7%	120	33.7%	0.7721 (0.5775-1.032)	0.08095
		T/C-C/C	267	56.8%	228	64.0%		
	Recessive	T/T-T/C	388	82.6%	301	84.6%	1.007 (0.6688-1.516)	0.9739
		C/C	61	13.0%	47	13.2%		
	Additive	–	–		–		0.8771 (0.7127-1.079)	0.2155

Some STAT3-SNP genotypes appeared to be more frequent in HC than in TB. Virtually, the frequency of rs2293152 genotype GG in HC subjects was significantly higher than that in TB patients compared to genotype CC/CG (**Table 3**, OR (95% CI) = 0.6586 (0.4657–0.9314), P = 0.01819), as analyzed by the Recessive model. Such statistical significance for GG versus CC/CG comparison between HC and TB was also revealed by Chi-squared distribution analysis (X2 = 5.619, P = 0.01776). In addition, the frequency of rs1053005 genotype AG/GG in HC subjects was higher than that in TB patients compared to genotype TT using both the Dominant model (**Table 3**, OR (95% CI) = 0.7426 (0.5622–0.9808), P = 0.03603) and the Chi-squared distribution (X2 = 4.404, P = 0.03585), respectively. The frequency of rs1053004 genotype TC in HC subjects was also significantly higher than that in TB patients compared to genotype TT and CC (**Table 3**, OR (95%CI) = 0.6995 (0.5113–0.957), P = 0.02542), as analyzed by the Codominant model. Furthermore, the frequency of allele CC of rs1053004 in HC control was significantly higher than that in TB patients using both the Additive model (**Table 3**, OR(95%CI) = 0.7781 (0.6286–0.9632), P = 0.0211) and the Chi-squared distribution (X2 = 5.319, P = 0.0211), respectively. Thus, the above SNP genotypes were more frequent in HC than in TB, suggesting that they were associated with healthy status, but not TB.

Interestingly, we found that two selected genotypes in the 2 STAT3-SNP loci rs1053004 and rs1053005 were associated with TB status. In fact, we found that the frequency of rs1053004 genotype TT in TB patients was significantly higher than that in HC subjects compared to genotype TC/CC, as analyzed by both the Dominant model (**Table 3**, OR (95% CI) = 0.694 (0.5175–0.9307), P = 0.01469) and the Chi-squared distribution analysis (X2 = 5.972, P = 0.01454), respectively. Moreover, rs1053005 genotype AA was significantly more frequent in TB than in HC compared to genotype AG/GG, as analyzed by the Dominant model (**Table 3**, OR (95%CI = 0.7426 (0.5622–0.9808), P = 0.03603). In contrast, none of the genotypes in the other two loci rs2293152 and rs744166 were significantly higher in TB patients than in HC, suggesting that these two STAT3-SNP loci were not associated with TB status.

To assess STAT3 SNP haplotypes for correlation with TB, we analyzed the linkage disequilibrium (**Table 4**) using the method as previously reported (Boulling et al., 2015). We found that the frequency of rs1053004–rs1053005 T-A haplotype in TB patients was significantly higher than that in HC (**Table 4**, OR 1.2989, 95%CI 1.0489–1.6086, P = 0.0165). The T-G haplotype was less frequent in TB patients compared with healthy controls (**Table 4**, OR 0.7855, 95% CI 0.6306–0.9784, P = 0.0312). Notably, the SNP rs1053004 locus is located in chromosome 17:42314074 and is close to the rs1053005 locus mapped to chromosome 17: 42313892.

**Table 4:** The association of haplotypes with the risk of tuberculosis.

Hap	CHR	SNPS	HAPLOTYPE	case_F	control_F	OR	95%CI	P-value
**STAT3**	17	rs1053004;rs1053005	TG	27 (0.033)	25 (0.038)	0.8819	0.5068-1.5345	0.6565
**STAT3**	17	rs1053004;rs1053005	CG	239 (0.294)	231 (0.347)	0.7855	0.6306-0.9784	0.0312
**STAT3**	17	rs1053004;rs1053005	TA	545 (0.671)	407 (0.611)	1.2989	1.0489-1.6086	0.0165

Our results therefore suggest that while STAT3-SNP rs1053004 and rs1053005 loci exhibited linkage disequilibrium, rs1053004 TT and rs1053005 AA genotypes or the T-A haplotype were associated with increased susceptibility to TB.

### STAT3 SNP rs1053004 TT and rs1053005 AA Genotypes Each Correlated With Severity of TB

**Figure 1:**
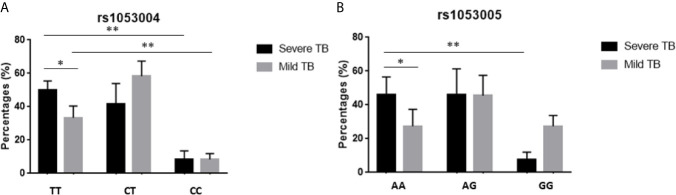
The percentages of subjects with different genotypes in TB patients. The bars showed the percentages of subjects with genotypes TT, CT and CC of rs1053004 **(A)**, and with AA, AG and GG of rs1053005 **(B)** in mild TB and severe TB, respectively. Data from 110 mild TB patients and 130 severe TB patients. *P < 0.05 and **P < 0.01.

We then examined whether the rs1053004 or rs1053005 genotype correlated with severe TB. To this end, we compared frequencies of rs1053004 and rs1053005 genotypes between patients with a mild form of TB and those with a severe form of TB. We focused on the rs1053004 TT genotype and the rs1053005 AA genotype, as these two STAT3-SNP loci each were associated with TB. Consistently, the rs1053004 TT genotype was significantly more frequent in severe TB than that in mild TB patients (**Figure 1A**). Similarly, the rs1053005 AA genotype was more frequent in severe TB than mild TB (**Figure 1B**). In contrast, the rs1053004 CT genotype and rs1053005 GG genotype were each more frequent in mild TB than in severe TB (**Figures 1A, B**). These results suggest that the rs1053004 TT and the rs1053005 AA genotypes not only were associated with susceptibility to TB but also correlated with TB severity.

### The rs1053005 AA Genotype Coincided With Low Constitutive Expression of *stat3* and *IL-17A* in PBMC, and the Variant *stat3* of rs1053004–rs1053005 T-A Haplotype Indeed Reduced *stat3* Expression in Reporter Assays

**Figure 2:**
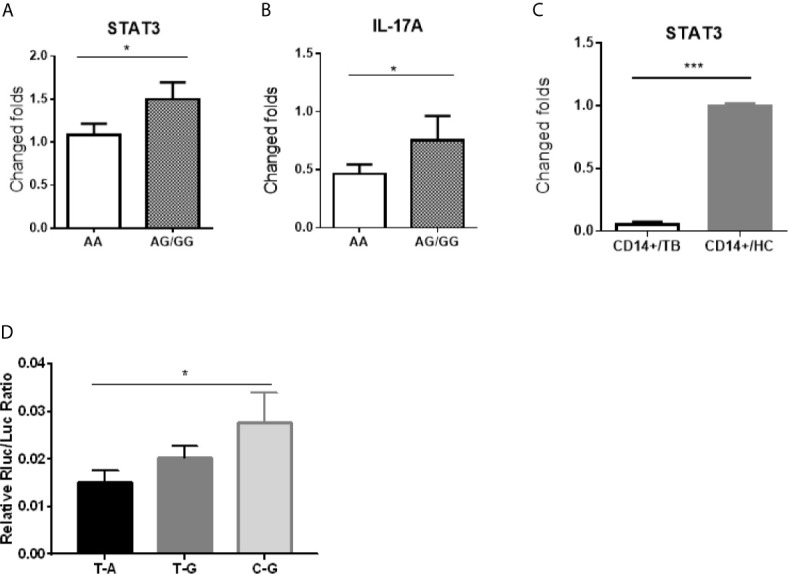
The *stat3* rs1053005 AA genotype coincided with the reduced constitutive expression of *stat3* and *IL-17A* in PBMC, and the variant *stat3* of rs1053004–rs1053005 T-A haplotype indeed resulted in a reduced *stat3* expression in reporter assays. Shown in **(A, B)** were expression levels of *stat3* and IL-17A RNA, respectively, in PBMC of uninfected healthy control (HC) subjects expressing genotype AA (84 subjects) and AG or GG (AG/GG, 72 subjects). *Stat3* and IL-17A RNA expressions were measured by qRT-PCR using PBMC freshly isolated from HC donors, and plotted as fold changes based on *stat3* expression in genotype AA subjects. **(C)** shows comparative expression of *stat3* RNA in CD14+ cells isolated from 30 HC subjects and 40 TB patients who were associated with the AA genotype or T-A haplotype. The *stat3* expressions were measured by qRT-PCR and plotted as relative expression as described above. **(D)** shows that the variant *stat3* of rs1053004–rs1053005 T-A haplotype indeed resulted in a reduced *stat3* expression in reporter assays. A luciferase encoding gene expression system was used to determine if the variant *stat3* RNA of rs1053004–rs1053005 T-A haplotype of *stat3* 3’UTR could influence the expression of *stat3* itself. 293T cells were transformed with plasmids containing SNP rs1053004–rs1053005 haplotypes T-A, T-G and C-G, respectively, and were tested for luciferase activity. Data are means ± standard errors. *P < 0.05 and ***P < 0.001.

Because the SNP rs1053004 and rs1053005 loci both locate in 3’-UTR of the *stat3* gene, these *stat3* variants were anticipated to influence the STAT3 gene expression. From a gene-regulation standpoint, the *stat3* RNA structure of rs1053004 TT or rs1053005 AA genotype may directly impact the *in vivo* STAT3 expression in those TB patients. To address this, we comparatively measured *stat3* expression in PBMC between the HC subjects who exhibited rs1053005 AA genotype and those who displayed AG or GG genotype. Interestingly, we found that *stat3* expression in PBMC of HC subjects carrying the AA genotype was significantly lower than that in PBMC of subjects displaying AG or GG (AG/GG) genotype (p <0.05) (**Figure 2A**). Surprisingly, the constitutive *IL-17A* expression in PBMC of HC subjects carrying the AA genotype was also significantly lower than that of those subjects displaying AG or GG genotype (p <0.05) (**Figure 2B**). This *in vivo* finding appeared to be consistent with the scenario that STAT3 regulates IL-17A expression (Khader et al., 2007; Shen and Chen, 2018). We and others previously reported lower *stat3* expression in protective CD4+ T cells and γδ T cells in PBMC of TB patients (Shen et al., 2002; Bandaru et al., 2014). In the current study, we established that TB patients correlated with STAT3 SNP rs1053005 AA/rs1053004 TT genotypes. Thus, the correlation between low *stat3* in PBMC of HC and the rs1053005 AA genotype appeared to be in line with reduced *stat3* expression in CD4+ T and γδ T cells in PBMC of TB patients, as we and others previously published (Bandaru et al., 2014; Shen et al., 2017). To extend these findings, we examined whether TB also coincided with altered *stat3* expression in Mtb-targeted cells, CD14+ monocytes/macrophages in PBMC. We found that CD14+ monocytes/macrophages isolated from TB patients, who were associated with the AA/TT genotypes, expressed only ~10% of the *stat3* level as seen in HC (**Figure 2C**). The findings suggest that TB-associated STAT3 SNP AA genotype correlated with a reduced *stat3* expression in PBMC containing CD14+ monocytes/macrophages, CD4+ T and γδ T cells.

We then sought to test the hypothesis that *stat3* RNA structure derived from SNP genotype/haplotype at 3’-UTR ultimately leads to a reduced *stat3* expression. We already showed that the SNP rs1053004 TT and rs1053005 AA genotypes were in linkage disequilibrium, and that rs1053004/rs1053005 TT/AA genotypes and/or T-A haplotype were associated with TB. We therefore took advantage of these findings to examine whether variant *stat3* RNA structure derived from rs1053004–rs1053005 T-A haplotype led to a decreased expression of *stat3* itself. To this end, we exploited the double-luciferase reporter STAT3 expression system, as recently described (Wang et al., 2019). We constructed the STAT3 expression system by transfecting cells with expression plasmids recombined with the *stat3* 3’-UTR variants of rs1053004–rs1053005 T-A, T-G and C-G haplotypes, respectively. We then measured the luciferase activities of these transfected cells. The results showed that 293T cells transfected with the reporter plasmid carrying the T-A haplotype exhibited significantly lower relative luciferase activities than those with the C-G haplotype (**Figure 2D**), suggesting that *stat3* rs1053004–rs1053005 T-A haplotype in 3’-UTR could reduce the *stat3* gene expression.

Together, our results suggest that the TB-associated rs1053005 AA genotype coincided with the reduced expression of *stat3* and *IL-17A* in PBMC, and the rs1053004–rs1053005 T-A haplotype at 3’-UTR indeed resulted in a reduced *stat3* expression due to the variant *stat3* RNA structure in the reporter expression system.

### Host PBMC Expressing rs1053005 AA Genotype and Low Constitutive *stat3* Exhibited the Reduced Ability to Mount Fast-Acting Innate Immunity Against Mycobacterial Infection in Cellular Model

We already established that HC subjects carrying SNP rs1053005 AA genotype coincided with the reduced constitutive *stat3/IL-17A* expression and that the rs1053004–rs1053005 T-A haplotype indeed resulted in a reduced *stat3* expression due to the variant *stat3* RNA structure. To facilitate explanation of *stat3* SNP-associated susceptibility to TB/TB severity, we determined whether humans carrying rs1053005 AA genotype and reduced *stat3* expression exhibited a reduced ability to mount fast-acting innate immunity against TB infection. For proof-of-concept, we transiently co-cultured both the isolated PBMC and the BCG-infected A549 cells as a fast-acting innate immunity model and tested the ability of innate PBMC to limit/control intracellular mycobacterial infection from infected A549 lung cells. Use of BCG-infected cells, instead of direct Mtb exposure to PBMC, would optimize better control of individual variations of BCG uptake. Thus, PBMC containing monocytes/macrophages and γδ T cells (representative of innate-like cell populations) were isolated from uninfected HC who expressed the *stat3* rs1053005 AA, AG and GG genotypes, respectively. The isolated PBMC were then co-cultured for 3 days with BCG-infected A549 lung-epithelial cells, and then assessed for CFU counts in lysate of co-cultured cells. The use of BCG, not Mtb, for transient intracellular infection of lung cells/monocytes was justified, because published studies demonstrated that BCG was similar to Mtb in transient short-term (3-day) infection or replication in monocytes/macrophages (Worku and Hoft, 2000; Yang et al., 2019). Such 3-day infection of A549 and monocytes/macrophages (acquired from A549) in co-culture allowed us to evaluate fast-acting innate immunity components including macrophage antimicrobial activities and γδ T-mediated anti-mycobacterial immunity in the cellular model.

**Figure 3:**
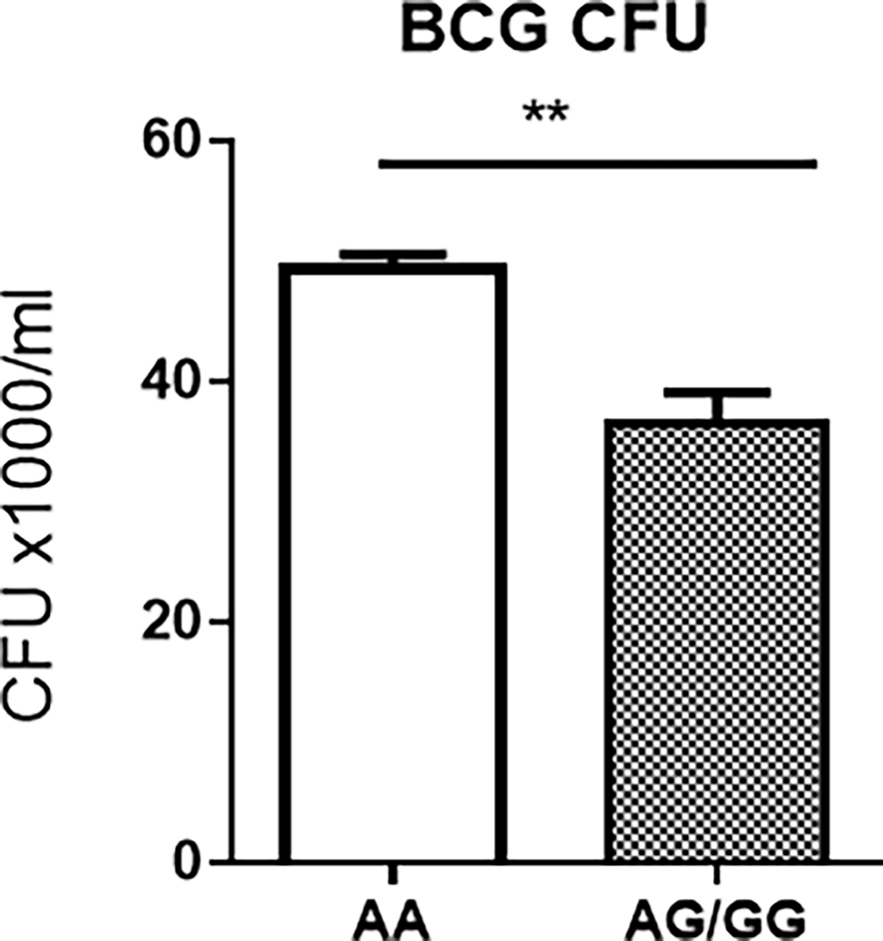
Host PBMC expressing the rs1053005 AA genotype and low constitutive *stat3* exhibited the reduced ability to mount fast-acting innate immunity against mycobacterial infection in cellular models. The graph shows that mean BCG CFU counts in co-cultures of BCG-infected A549 cells and PBMC isolated from uninfected HC expressing the *stat3* SNP AA genotype and low *stat3* were significantly higher than those displaying the AG (25 subjects) or GG (AG/GG, 42 subjects) genotype. The isolated PBMC were co-cultured for 3 days with BCG-infected A549 cells (6 h of MOI = 10) at the 10:1 ratio of PBMC: A549, and then the lysates of the co-cultures were assessed for BCG CFU counts. The co-culture system allowed us to minimize BCG-uptake/infection variations among tested individuals and to assess fast-acting innate immunity against mycobacterial infection in cellular models (see *Results*/*Discussion*). Data are means ± standard errors. **P < 0.01.

Surprisingly, BCG CFU counts in co-cultures from PBMC of uninfected HC expressing the *stat3* SNP AA genotype and low *stat3* were significantly higher than those displaying the AG or GG (AG/GG) genotype (**Figure 3**). Given the innate PBMC inhibition of mycobacteria in A549 cells and in monocytes/macrophages spread from infected A549 cells, we interpreted growth inhibition as fast-acting innate anti-mycobacterial immunity in cellular models. The results in our innate immunity model implied that the AA genotype/low STAT3 reduced the ability of innate populations in PBMC to mount fast-acting cellular immunity against intracellular mycobacterial infection.

Results from these in-depth mechanistic experiments support our hypothesis that *stat3* SNP AA genotypes and the low constitutive *stat3* expression reduce the ability of host innate cell populations to mount fast-acting immune defense against mycobacterial infection.

### STAT3 Down-Regulation Depressed Diverse Antimicrobial Activities Involving VDR-Related CYP27B1, DEFB4A and CAMP Pathways as Well as IL-32, iNOS and Autophagy Mechanisms, and Led to an Enhanced Mycobacterial Infection

Finally, we conducted additional in-depth mechanistic experiments to examine mechanisms whereby variant *stat3* RNA structure derived from SNP AA genotype at 3’-UTR, low constitutive *stat3* expression can depress STAT3-downstream pathways of fast-acting innate immunity against mycobacterial infection. Our above experiments already demonstrated that *stat3* SNP AA genotypes and the low constitutive *stat3* expression reduce the ability of host innate cell populations to mount fast-acting immune defense against mycobacterial infection.

Based on these results, we determined if down-regulation of STAT3 perturbed potential STAT3 downstream pathways of antimicrobial responses in target cells, promoting mycobacterial infection. It has been well known that one STAT3 downstream pathway activates Th17/Th22 differentiation in anti-TB immune responses of T cells (Wang et al., 2013). However, it remains unknown what other undefined STAT3 downstream pathways can also involve antimicrobial activity or fast-acting innate-like immunity against mycobacterial infection (Queval et al., 2016; Arcos et al., 2017). Given the possibility that Vitamin D receptor (VDR)-related CYP27B1, DEFB4A and CAMP pathways involve not only macrophages but also innate-like γδ T cells or others, we tested the hypothesis that low STAT3 broadly inhibit CYP27B1, DEFB4A and CAMP pathways, leading to an enhanced mycobacterial infection.

**Figure 4:**
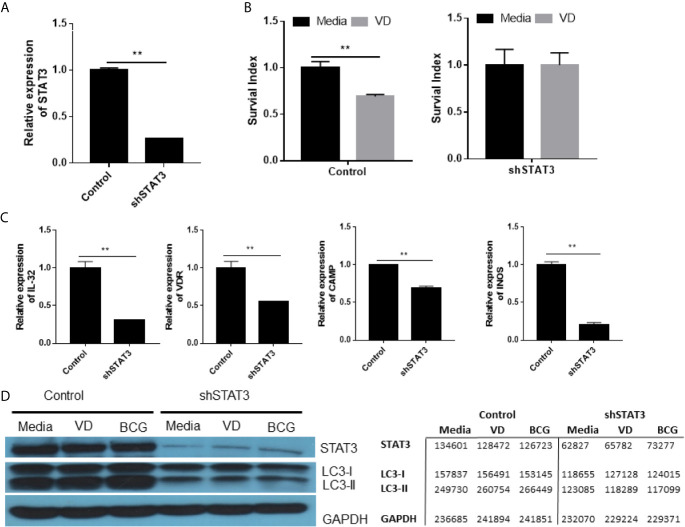
STAT3 down-regulation depressed diverse antimicrobial activities involving VDR-related CYP27B1, DEFB4A and CAMP pathways as well as IL-32, iNOS and autophagy mechanisms, and led to an enhanced mycobacterial infection. **(A)** shows STAT3 down-regulation in A549 cells after transfection with the plasmid encoding STAT3 shRNA. **(B)** shows that the Vitamin D (VD) treatment of BCG-infected A549 cells transfected with plasmid control mediated inhibition of BCG growth (left panel), whereas STAT3 down-regulation in A549 cells transfected with the shRNA reduced the VD-mediated inhibition of BCG infection (right panel). Shown was the percentage growth index representing intracellular bacteria survival calculated as follows: Growth Index = 100 × CFU of treatment/CFU of media. **(C)** shows that STAT3 down-regulation by the shRNA transfection into A549 decreased expressions of gene encoding IL-32, VDR, CAMP, and iNOS during BCG infection, compared with controls. **(D)** shows representative results in Western blot (WB) indicating that STAT3 down-regulation by the shRNA indeed reduced protein expressions of STAT3, IC3 I and II in the settings of media control, VD treatment and BCG infection, respectively. The values of band densities in WB figure were measured by ImageJ software and shown in right. Data are means ± standard errors. **P < 0.001.

To knock-down or silence STAT3 expression, we transduced cells with lentivirus expressing shRNA of *stat3* gene (shSTAT3). This would provide an alternative approach to circumvent the unavailability of *stat3* knockout mice due to the crucial role of STAT3 in embryogenesis (Takeda et al., 1997a). Our shSTAT3 approach reproducibly silenced or knocked-down *stat3* expression by ~80% compared to the control (**Figure 4A**), and consistently decreased STAT3 protein production in shSTAT3-transduced cells as shown in western blot assay (**Figure 4D**). These results demonstrated that shSTAT3 could successfully knock down or silence STAT3 expression.

Using this shSTAT3 approach, we first demonstrated that silencing STAT3 reduced the vitamin D (VD)-mediated inhibition of intracellular BCG growth in the transduced cells compared to the control (**Figure 4B**). Then, we sought to determine if silencing STAT3 by shSTAT3 could reduce IL-32 expression altering innate antimicrobial response. To date, it remains unknown whether STAT3 can activate the IL-32 pathway (Pham et al., 2019), although IL-32 was reported to mediate IFN-γ- and VD-driven antimicrobial activity (Montoya et al., 2014) and induce expression of iNOS for NO inhibition of intracellular mycobacteria (Zhou and Zhu, 2015). We found that silencing STAT3 by shSTAT3 led to ~75% reduction of IL-32 expression compared to the control (**Figure 4C**), suggesting that STAT3 signaling can indeed activate IL-32 expression. Because IL-32 signaling can activate VD and NO antimicrobial pathways, we determined whether silencing STAT3 caused changes in key genes involving these two pathways. We found that silencing STAT3 by shSTAT3 significantly decreased the expressions of VDR, CAMP, and iNOs compared to the control during BCG infection (**Figure 4C**). These results indicate that reduction in STAT3 expression could depress IL-32-driven VD and NO antimicrobial pathways.

Furthermore, we determined whether silencing STAT3 could impact autophagy pathway. The ability of STAT3 to regulate autophagy has not been reported (Upadhyay et al., 2018), although autophagy acts as an important part to eliminate intracellular microorganisms by lysosomal degradation (You et al., 2015). Here we examined whether the shSTAT3-induced silencing of STAT3 could decrease the expression of LC-3II, an essential processed form of autophagy response against microbes. We found that silencing STAT3 by shRNA remarkably decreased the production of LC-3II protein even in the VD stimulation or BCG infection (**Figure 4D**). In fact, the LC-3II protein was less abundant than LC-3I (**Figure 4D**), suggesting that reduced expression of STAT3 indeed suppresses autophagy response in the presence of VD or BCG stimulation.

Thus, our results and in-depth mechanistic studies demonstrated that STAT3 down-regulation depressed diverse antimicrobial activities involving VDR-related CYP27B1, DEFB4A and CAMP pathways as well as IL-32, iNOS and autophagy mechanisms, leading to an enhanced mycobacterial infection.

## Discussion

STAT3 is an important transcriptional factor involved in a broad spectrum of biological functions (Li et al., 2014). Since germline deletion of STAT3 in mice results in an early embryonic lethality (Takeda et al., 1997), Cre-loxP recombination system to ablate the mouse STAT3 gene in later life emerged as a complex/unpractical tool to assess STAT3 for biological roles. However, in the setting of human diseases, STAT3 SNP analysis appears to be important and practical for studies of cancers and virus infections/diseases (Eto et al., 2013; Xie et al., 2013; Moazeni-Roodi and Hashemi, 2018; Lai et al., 2019). In the current study, we employed a combination of STAT3 SNP analysis and mechanistic experiments in humans and cellular models, because the identified STAT3 SNP and relevant functions can be evaluated and characterized in cellular models with gene-targeted manipulations including short-term STAT3 knock-down (Shen et al., 2017; Shen and Chen, 2018).

STAT3 SNP TT/AA genotypes and T-A haplotype appear to be a genetic risk factor predisposing humans to TB and TB severity. Our large-scale case-control studies of STAT3 SNP demonstrated that rs1053004 TT and rs1053005 AA genotypes or T-A haplotype were associated with an increased susceptibility to TB and severe TB. Similarly, other studies have shown that rs1053004–rs1053005 T-A haplotype were also associated with higher HBV DNA levels (Li et al., 2018). Given the diverse biological functions of STAT3 (Bharadwaj et al., 2020), further STAT3 SNP studies in other human infections may uncover that rs1053004 TT and rs1053005 AA genotypes or T-A haplotype could be a broad risk factor susceptible to diseases after infection.

Our data in humans and cellular models implicated that the STAT3 SNP AA genotype and T-A haplotype in 3’-UTR indeed reduced *stat3* RNA expression. In fact, rs1053005 AA genotype correlated with the reduced constitutive expression of STAT3 in PBMC of HC. And the AA genotype/T-A haplotype coincided with a reduced STAT3 expression in CD14+ monocytes/macrophages in PBMC from TB patients who significantly associated with the AA allele and T-A haplotype. These results were consistent with the reduced STAT3 expression in CD4+ T cells and γδ T cells of TB patients, as published by us and another group. Notably, our mechanistic experiments confirmed that the variant *stat3* RNA derived from rs1053004–rs1053005 T-A haplotype indeed reduced *stat3* expression perhaps due to the variant structure itself in the reporter gene-expression system. Our extensive findings are in line of the other report demonstrating that cancer patients with STAT3 SNP rs1053004 TT genotype expressed lower STAT3 protein as detected by Western blotting when compared to those with CC genotype (Lai et al., 2019).

Our in-depth mechanistic experiments also demonstrated for the first time that TB-associated STAT3 SNP AA genotype and low constitutive STAT3 led to a reduced ability of innate PBMC to control mycobacterial infection spread from BCG-infected lung cells in the cellular model. In general, the mycobacterial growth inhibition that we detected here in naïve PBMC from uninfected subjects mainly involved anti-mycobacterial activities of monocytes/macrophages and innate-like γδ T cells or other innate lymphocytes, because adaptive CD4+/CD8+ T cells in PBMC usually require *in vivo* priming or prior *in vitro* stimulation by antibodies or others in the purification process in the mycobacterial growth inhibition assay (Shen et al., 2017; Yang et al., 2019). Notably, because of huge labors for mycobacterial inhibition assays, we were unable to purify macrophages, γδ T cells and others, respectively, in our large-scale studies. We therefore used naïve PBMC isolated from subjects to detect anti-mycobacterial function of monocytes or representative innate-like γδ T cells. Based on the innate PBMC inhibition of mycobacterium BCG in A549 cells and in monocytes/macrophages spread from infected A549 cells, we interpreted the growth inhibition as fast-acting innate anti-mycobacterial immunity in cellular models. Recently, PBMC or whole blood has been widely employed in mycobacterial inhibition assays for human TB studies (Cheon et al., 2002; Lee et al., 2019). In fact, we found that the assay of PBMC co-culturing with BCG-infected A549 cells was practically achievable and reproducible.

To date, in-depth human studies have not been done to determine whether *stat3* SNP and STAT3 down-regulation can influence downstream innate immunity pathways or reduce host immunity against human TB. Our findings in the current study implicate that *stat3* SNP TT/AA genotypes or T-A haplotype to reduce STAT3 expression or signaling. We also demonstrated for the first time that such STAT3 down-regulation can depress downstream multiple anti-mycobacterial pathways of VDR-related CAMP pathway as well as IL-32, iNOS and autophagy mechanisms. It is likely that the *stat3* SNP and STAT3 down-regulation reduce both the ability of macrophages to exert the antimicrobial phagosome/NO killing and the capability of innate-like T cells/lymphocytes to mount the anti-mycobacterial immunity. Consequently, such reduced innate/innate-like immunity would compromise the development of adaptive immune response, leading to an enhanced mycobacterial infection or progression to TB. Thus, these complex interactions postulate consequences as follows: STAT3 SNP–STAT3 downregulation–downstream multiple anti-mycobacterial pathways–an enhanced mycobacterial infection (Verway et al., 2013; Kim et al., 2018).

In summary, our experimental studies in humans and cellular models provided previously unreported findings and functional mechanisms as follows: (i) *stat3* SNP rs1053004 TT and rs1053005 AA genotypes or T-A haplotype were associated with susceptibility to TB or TB severity; (ii) the rs1053005 AA genotype coincided with the reduced constitutive expression of *stat3* and *IL-17A* in PBMC, and the variant *stat3* of rs1053004–rs1053005 T-A haplotype indeed resulted in a reduced *stat3* expression in reporter assays; (iii) host PBMC expressing the rs1053005 AA genotype and low constitutive *stat3* exhibited the reduced ability to mount fast-acting innate immunity against mycobacterial infection in cellular models; (iv) the STAT3 down-regulation broadly depressed STAT3 downstream anti-mycobacterial activities involving VDR-related CAMP pathway as well as IL-32, iNOS and autophagy mechanisms, leading to an enhanced mycobacterial infection. Thus, the current study helps to establish the hypothetical regulatory axis of STAT3 SNP–STAT3 downregulation–downstream multiple anti-mycobacterial pathways in enhanced mycobacterial infection. Together, our findings suggest that low constitutive *stat3* derived from the AA genotype or T-A haplotype to down-regulate STAT3, then depress downstream multiple anti-mycobacterial pathways/mechanisms, and then lead to an enhanced mycobacterial infection or TB.

## Data Availability Statement

The original contributions presented in the study are included in the article/supplementary material. Further inquiries can be directed to the corresponding authors.

## Ethics Statement

The studies involving human participants were reviewed and approved by the Shanghai Pulmonary Hospital. The patients/participants provided their written informed consent to participate in this study.

## Author Contributions

FW, ZC, and HS conceptualized the study and conceived the project. FW, GH, and WS contributed with study design, sample processing and data analysis. LS contributed with data analysis. YP helped with sample processing. WS contributed with the recruitment of the participants and sample collection. ZC and HS wrote the paper with input from all other authors. All authors contributed to the article and approved the submitted version.

## Funding

This work was supported by the Chinese National Major Projects Grants [2018ZX10731301-006-001 to HS], National Natural Science Foundation of China Grants [81401711 to FW, 31970876 and 32070943 to HS], Shanghai National Natural Science Foundation of Grant [20ZR1406200 to FW], Medical Research Plan of Fudan University [DGF501022/028/002 to FW], and Clinical Research Plan of SHDC [16CR1028B to WS].

## Conflict of Interest

The authors declare that the research was conducted in the absence of any commercial or financial relationships that could be construed as a potential conflict of interest.
